# Supplementary figures and images for: Origin and evolution of the zinc finger antiviral protein
Source: PLoS Pathog. 2021 Apr 26;17(4):e1009545. doi: 10.1371/journal.ppat.1009545 (PMC8102003; doi:10.1371/journal.ppat.1009545)

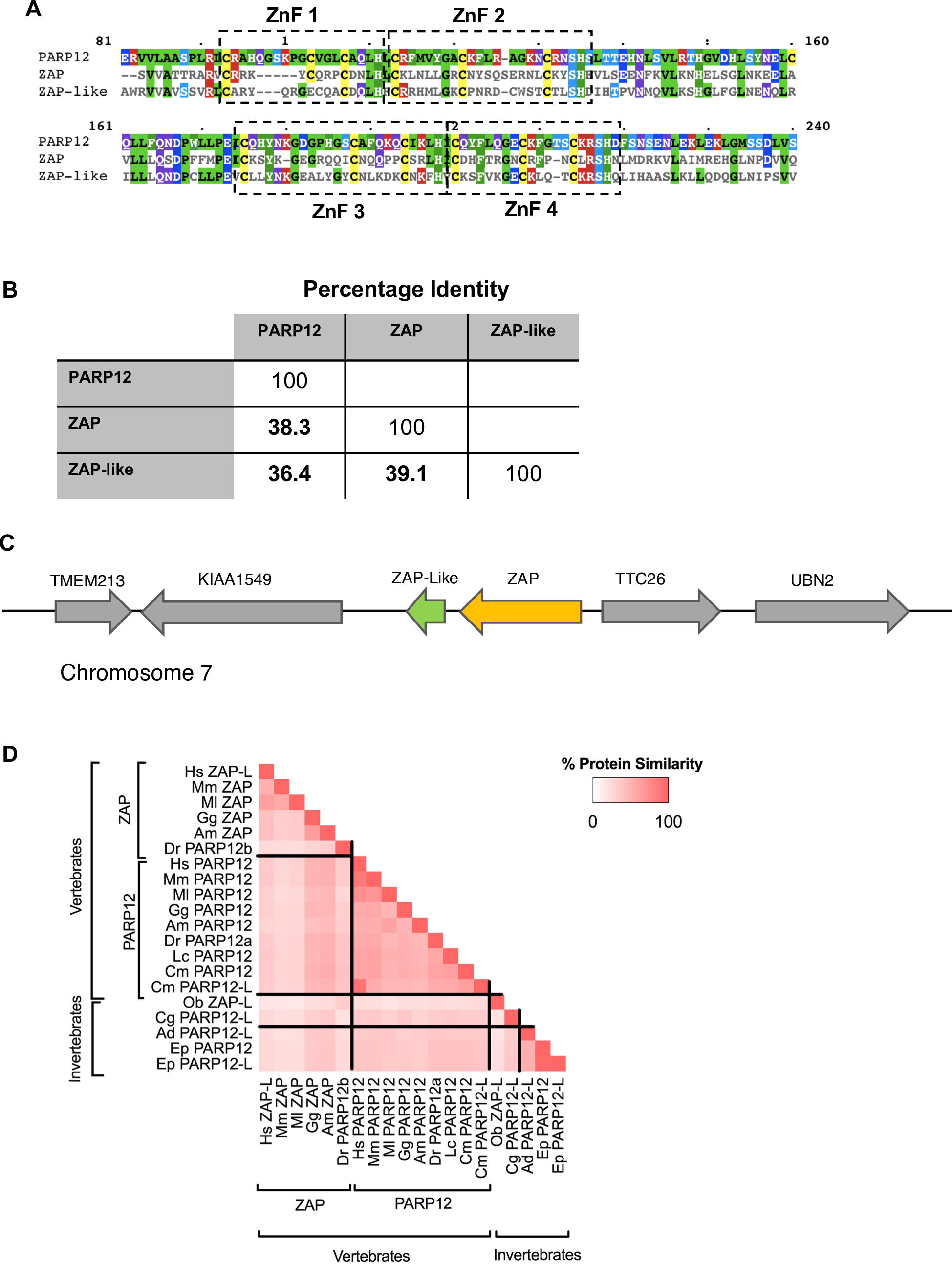

Supplement: S1 Fig — (A) Protein sequence alignment of the N-terminal domain (NTDs) of human ZAP, PARP12 and ZAP-like protein. Colored residues indicate amino acid properties and conservation. (B) Percentage identity matrix among human ZAP, PARP12 and ZAP-like proteins. (C) Locus surrounding human ZAP and ZAP-like genes in chromosome 7. (D) Percentage protein similarity one-to-one matrix among ZAP and PARP12 paralogues found in vertebrate and invertebrates species. Hs, Homo sapiens (human); Mm, Mus musculus (mouse); Ml, Myotis lucifugus (little brown bat), Gg, Gallus gallus (chicken); Am, Alligator mississipiensis (alligator); Dr, Danio rerio (zebrafish); Lc, Latimeria chalumnae (Coelacanth); Ob, Octopus bimaculoides (California two-spot octopus); Cg, Crassostrea gigas (oyster); Ad, Acropora digitifera (coral). (TIF) [file ppat.1009545.s001.tif]

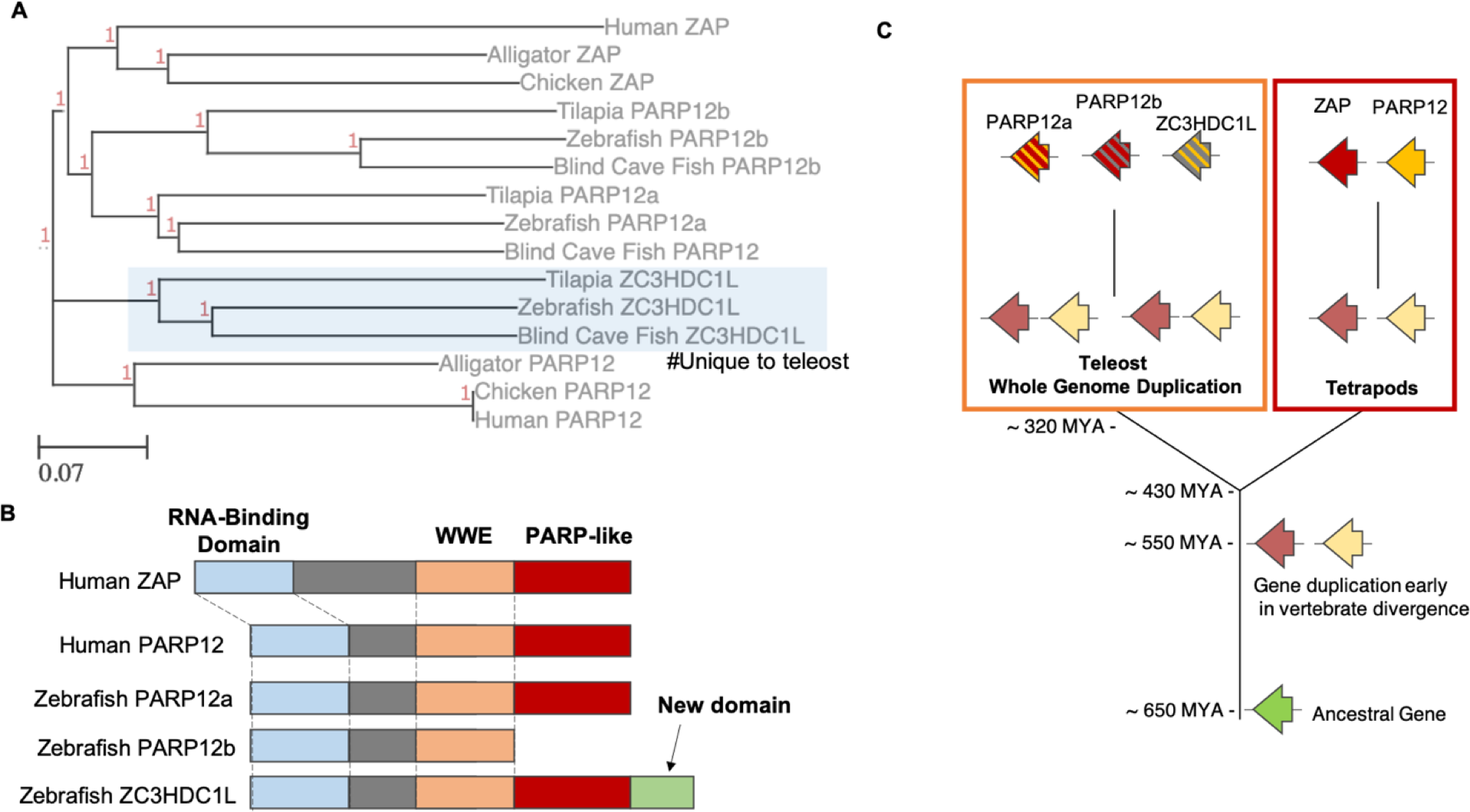

Supplement: S2 Fig — (A) Phylogenetic analysis of ZAP and PARP12-related sequences found in the genomes of tetrapods and teleost fish. Blue box marks cluster of sequences unique to teleost fish. (B) Schematic representation of the domain organization of human ZAP and zebrafish PARP12a, PARP12b and ZC3HD1CL. (C) Diagram of the proposed evolution of ZAP/PARP12-related genes in tetrapods and teleost fish. MYA, million years ago. (TIF) [file ppat.1009545.s002.tif]

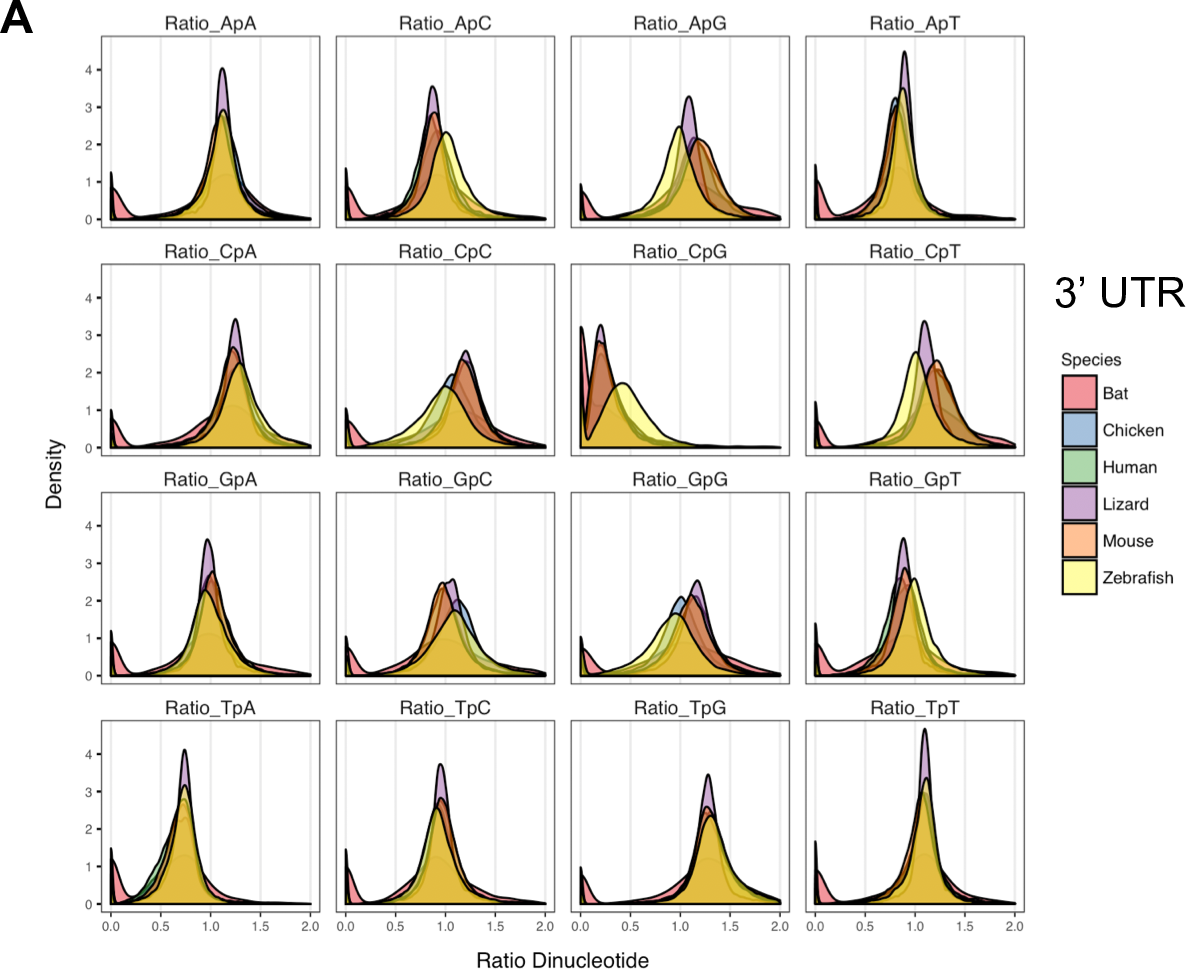

Supplement: S3 Fig — (A) The 3’ untranslated regions of mRNA transcripts found in transcriptomes of several vertebrates were collected from the NCBI nucleotide database and dinucleotide frequency ratio (observed/expected) was calculated and frequency distribution for each dinucleotide was plotted. (TIF) [file ppat.1009545.s003.tif]

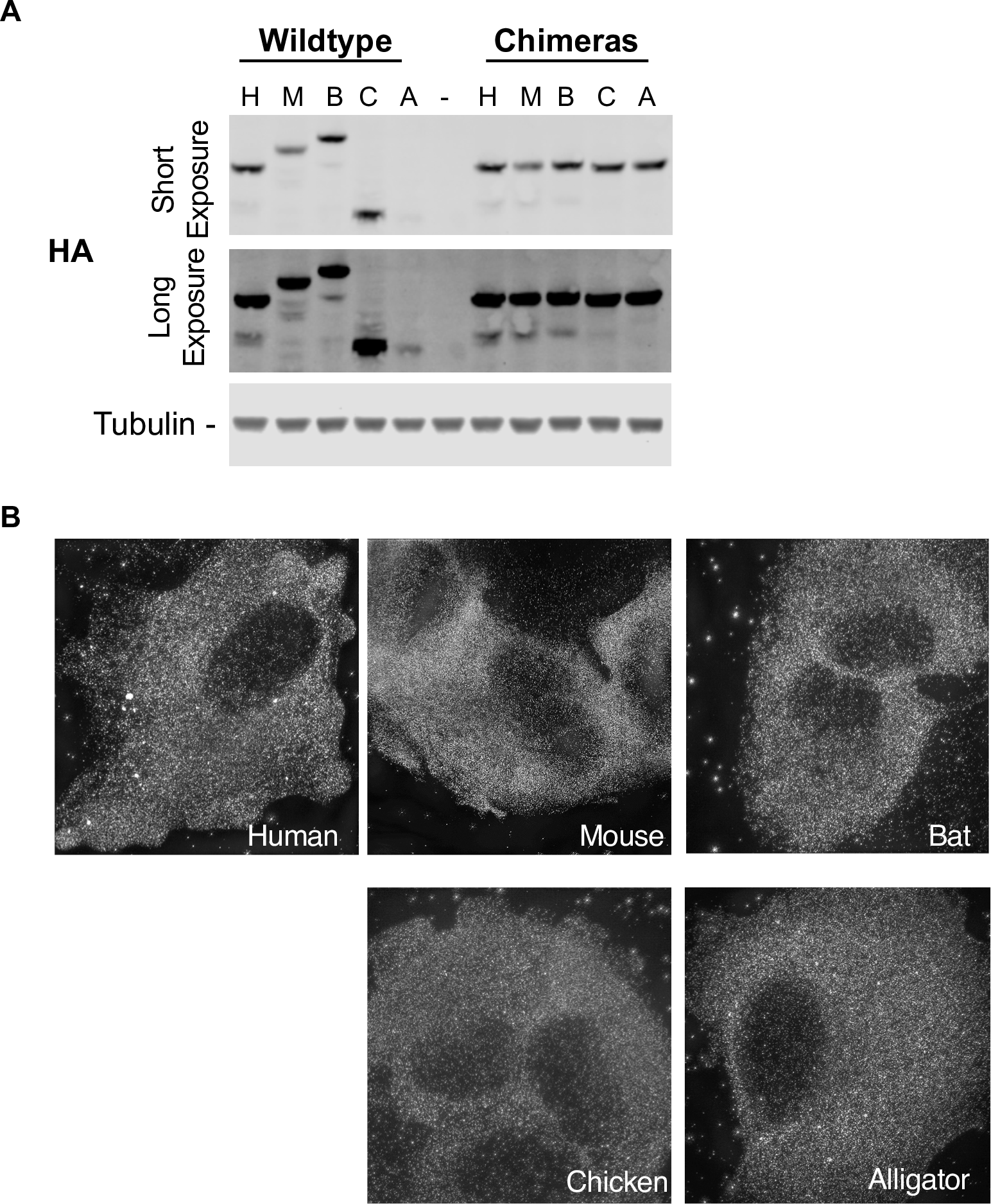

Supplement: S4 Fig — (A) HEK293T ZAP-/- cells were transfected with plasmids encoding wildtype or chimera ZAP from human (H), mouse (M), bat (B), chicken (C) or alligator (A). As a control, cells were transfected with an equivalent amount of an empty plasmid (-). After 48h, whole cell lysates were generated and analysed by SDS-PAGE/western blot. (B) HOS ZAP-/- cells were transduced with vectors encoding human ZAP or ZAP chimeras all C-terminally tagged with the HA epitope. Expression of ZAP was induced by treatment with doxycycline and 48h later cells were fixed, stained with an anti-HA antibody and immunofluorescent antibodies and imaged. Micrographs are representative of each imaged condition. (TIF) [file ppat.1009545.s004.tif]

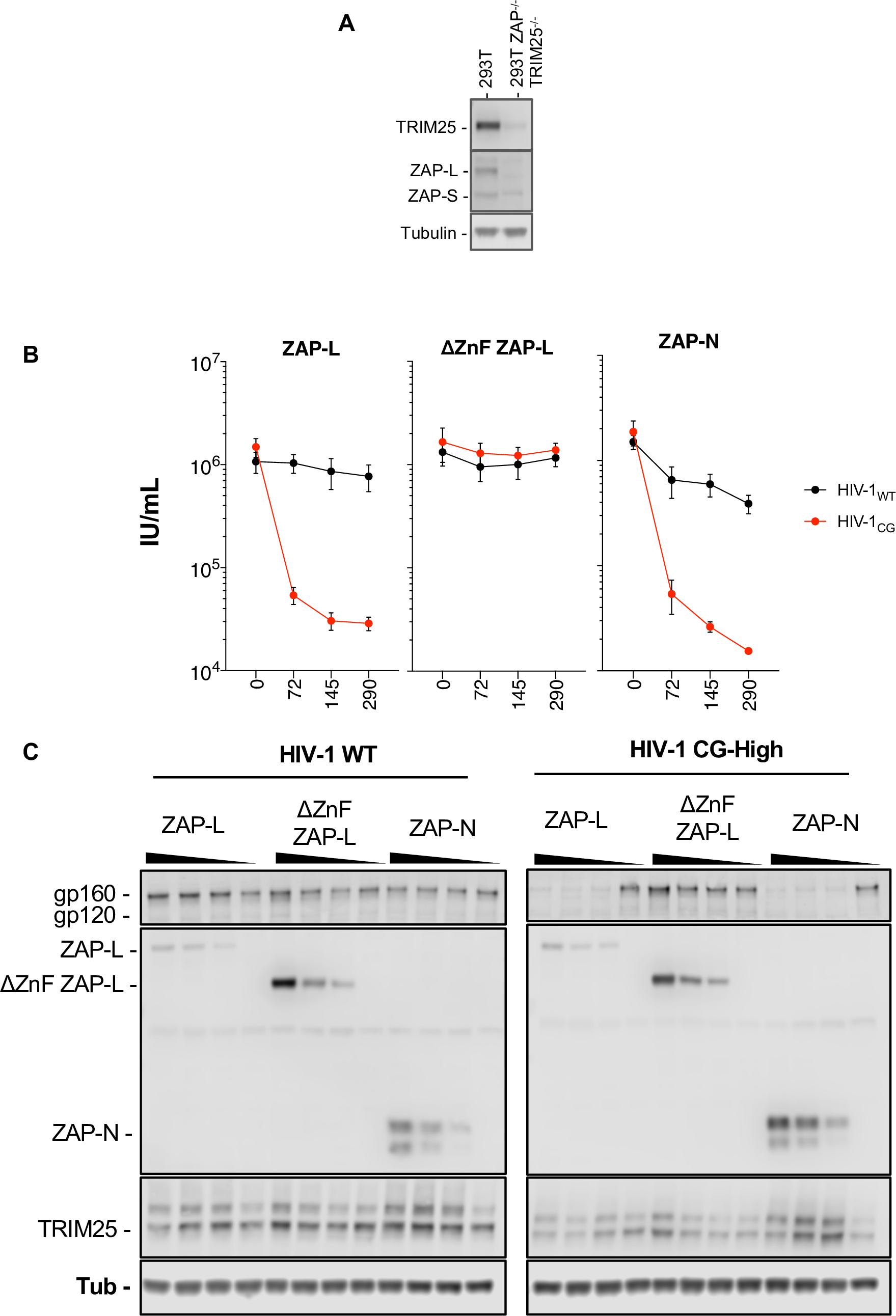

Supplement: S5 Fig — (A) Western blot analysis of HEK293T and HEK293T ZAP-/- and TRIM25-/-. (B and C) To test if the RNA-binding domain of ZAP was sufficient to inhibit the replication of HIV-1CG, we cotransfected HEK293T ZAP-/- cells with proviral plasmids encoding HIV-1WT or HIV-1CG and increasing amounts of plasmids (0, 72, 145 and 290 ng) encoding the full-length ZAP-L, a truncated form of ZAP lacking the RNA-binding domain (ΔZnF ZAP-L) and a truncated form of ZAP composed solely of the 254 N-terminal amino acids of ZAP (ZAP-N). After 48h, virus yield was measured and whole cell lysates were generated and analysed by SDS-PAGE and western blot. (TIF) [file ppat.1009545.s005.tif]

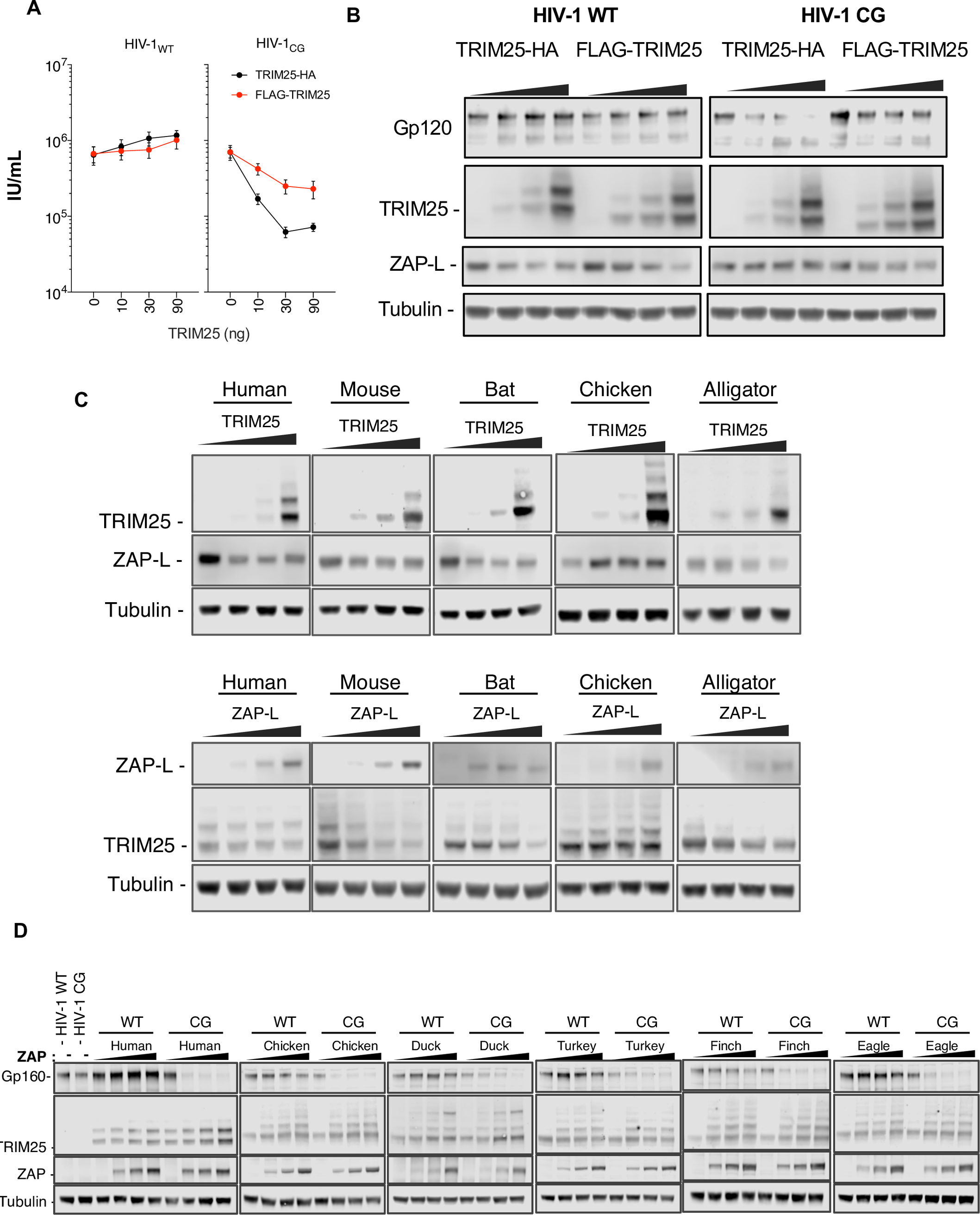

Supplement: S6 Fig — (A and B) To assess if terminal modifications of TRIM25 would impact its antiviral activity, we cotransfected HEK293T ZAP-/- and TRIM25-/- cells with proviral plasmids encoding either HIV-1WT or HIV-1CG along with increasing amounts (0, 10, 20 or 90 ng) of N-terminally tagged TRIM25 (FLAG-TRIM25) or C-terminally tagged TRIM25 (TRIM25-HA). Forty-eight hours after transfection, viruses were harvested and titred onto MT4-R5-GFP cells while whole cells lysates of producer cells were generated and analyzed by western blot. (C) To evaluate if over-expression of TRIM25 would affect the expression levels of ZAP, we have co-transfected plasmids encoding ZAP chimeras with increasing amounts of plasmids encoding their cognate TRIM25. Cells were lysed 48h post-transfection and protein complexes were resolved and analyzed by SDS-PAGE/western blot. (D) Cells were transfected with human ZAP or avian ZAP chimeras along with proviral plasmids, as indicated in the main text. After 48h, whole cell lysates were generated, resolved by SDS-PAGE and analyses by western blot. (TIF) [file ppat.1009545.s006.tif]

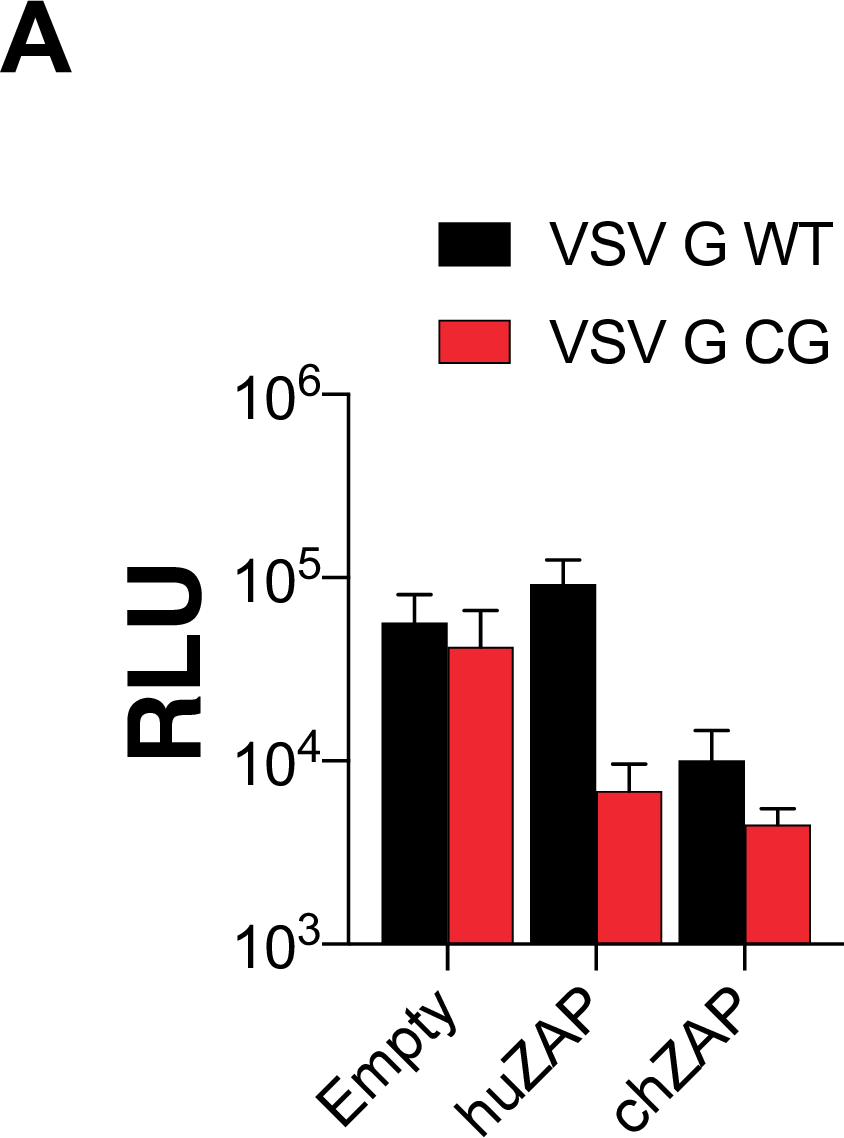

Supplement: S7 Fig — (A) HEK293T ZAP-/- and TRIM25-/- were co-transfected with luciferase reporters containing, as 3’UTRs, VSV-G wildtype sequences or CpG-enriched VSV-G sequences and plasmids encoding human and chicken ZAP and TRIM25. After 56h, cells were lysed and luciferase activity was measured. RLU, Relative light units. (TIF) [file ppat.1009545.s007.tif]

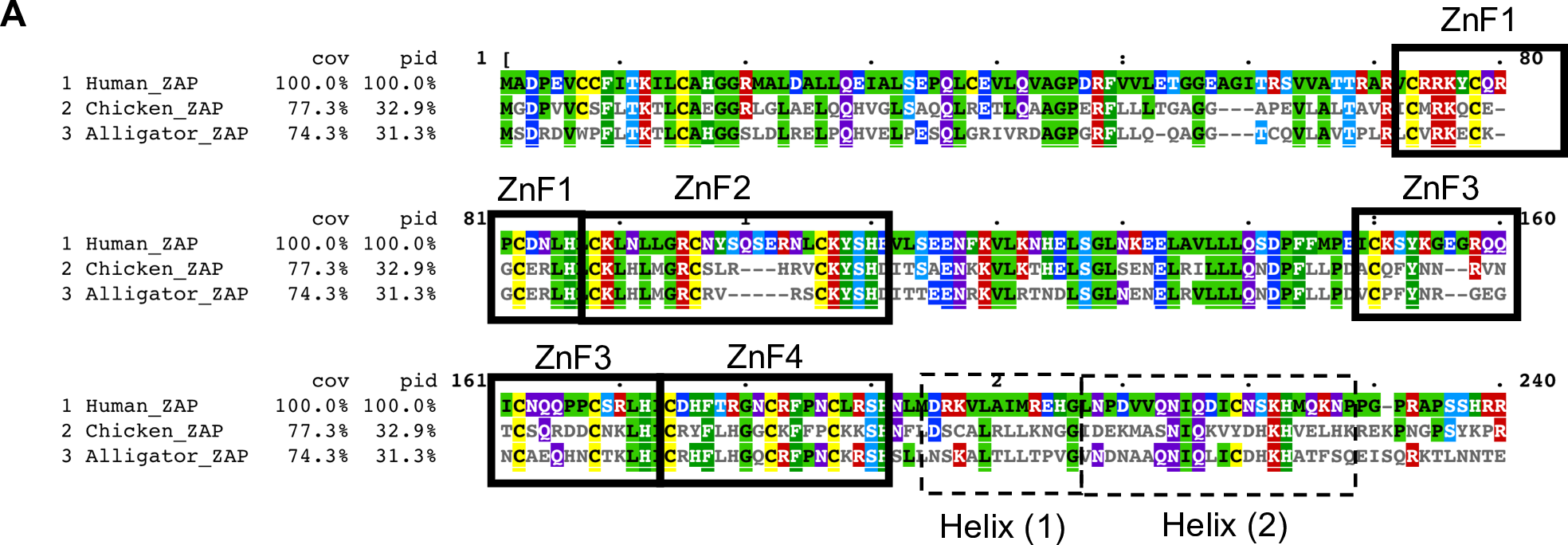

Supplement: S8 Fig — (A) Protein sequence alignment of human, chicken and alligator N-terminal region. Zinc fingers are highlighted in solid line boxes while putative helices (1) and (2) are highlighted in dashed line boxes. Cov, percentage of sequence coverage; Pid, percentage of protein identity. (TIF) [file ppat.1009545.s008.tif]
